# EBI2 Is a Negative Regulator of Type I Interferons in Plasmacytoid and Myeloid Dendritic Cells

**DOI:** 10.1371/journal.pone.0083457

**Published:** 2013-12-26

**Authors:** Eugene Y. Chiang, Robert J. Johnston, Jane L. Grogan

**Affiliations:** Department of Immunology, Genentech Inc., South San Francisco, California, United States of America; Kantonal Hospital St. Gallen, Switzerland

## Abstract

Epstein-Barr virus induced receptor 2 (EBI2), a Gα_i_-coupled G protein-coupled receptor, is a chemotactic receptor for B, T and dendritic cells (DC). Genetic studies have also implicated EBI2 as a regulator of an interferon regulatory factor 7 (IRF7)-driven inflammatory network (IDIN) associated with autoimmune diseases, although the corollary in primary type I IFN-producing cells has not been reported. Here we demonstrate that EBI2 negatively regulates type I IFN responses in plasmacytoid DC (pDCs) and CD11b^+^ myeloid cells. Activation of EBI2^−/−^ pDCs and CD11b^+^ cells with various TLR ligands induced elevated type I IFN production compared to wild-type cells. Moreover, *in vivo* challenge with endosomal TLR agonists or infection with lymphocytic choriomeningitis virus elicited more type I IFNs and proinflammatory cytokines in EBI2^−/−^ mice compared to normal mice. Elevated systemic cytokines occurred despite impaired ability of EBI2-deficient pDCs and CD11b^+^ cells to migrate from the blood to the spleen and peritoneal cavity under homeostatic conditions. As reported for other immune cells, pDC migration was dependent on the ligand for EBI2, 7α,25-dihydroxycholesterol. Consistent with a cell intrinsic role for EBI2, type I IFN-producing cells from EBI2-deficient mice expressed higher levels of IRF7 and IDIN genes. Together these data suggest a negative regulatory role for EBI2 in balancing TLR-mediated responses to foreign and to self nucleic acids that may precipitate autoimmunity.

## Introduction

Type 1 IFNs are a group of pleiotropic cytokines that are important for protection against viral infections; however, dysregulated type I IFN responses may precipitate and perpetuate autoimmune diseases [1]. Accordingly, the signaling pathways involved in type I IFN production must be tightly regulated, involving control mechanisms at multiple levels, including adaptor complex destabilization, phosphorylation and ubiquitination of signal proteins and transcriptional regulation [2], [3]. During acute viral infections, a rapidly induced transient burst of type I IFN is produced [4]. While IFN-β can be produced by most cell types, including dendritic cells (DCs), macrophages and epithelial cells, the primary source of IFN-α is the plasmacytoid DC (pDC) [5]–[7]. Recognition of viruses and subsequent elaboration of type I IFN responses is dictated in large part by TLR, principally the nucleic acid-sensing TLR expressed in endosomes: TLR3, TLR7, TLR8 and TLR9 [8], [9]. While these TLRs are all expressed in DCs, macrophages and B cells, pDCs exclusively express high constitutive levels of TLR7 and TLR9 which recognize guanosine/uridine-rich ssRNA and dsDNA rich in unmethylated CpG sequences, respectively, contributing to their specialized role in antiviral defense [10], [11]. pDCs are additionally primed to mount potent type I IFN responses due to their high basal expression of the transcription factor IRF7, the master regulator of type I IFN responses [12]. IRF7 resides in the cytoplasm as a “latent” form, but is phosphorylated and activated upon MyD88-dependent TLR7/9 signaling [13]. Inherent to using TLRs to detect viral pathogens is the risk of recognition of self nucleic acids [10]. Roles for IFN-α and pDCs in type 1 diabetes (T1D) have also been demonstrated [14], [15], and IFN gene signatures have been described in rheumatoid arthritis (RA), multiple sclerosis (MS), psoriasis and Sjogren’s syndrome [16]–[22].

Epstein-Barr virus-induced receptor 2 (EBI2), a Gα_i_-coupled G protein-coupled receptor (GPCR) [23], has been described as a chemotactic receptor for B cells and splenic DC, particularly the CD4^+^ DC subset [24]-[29]. EBI2 expression has been well characterized for B cells, where differential expression of EBI2 during B cell maturation is a key regulator of B cell positioning in lymphoid follicles, collaborating with other B cell-expressed chemokine receptors including CXCR5 and CCR7 [24], [25], [30]. The migration of B cells is dictated by the oxysterol 7α,25-dihydroxycholesterol (7α,25-OHC) [26], [27], [31], thereby ascribing EBI2 with a functional role as a chemotactic receptor. Moreover, EBI2 is required for positioning splenic CD4^+^ DC into bridging channels within germinal centers, which may promote sampling of systemic, particulate antigens [28], [29]. Intriguingly, EBI2 is expressed by many cell types involved in immune responses, including CD4^+^ T cells, a subset of CD8^+^ T cells, NK cells, DCs, macrophages and neutrophils, [23], [25]–[27], [29], [32] suggesting it may regulate positioning and function of a broad array of immune cell types.

In addition to its role as a chemotactic receptor, EBI2 has also been identified as a candidate for *trans*-regulation of the IFN regulatory factor 7 (IRF7)-driven inflammatory network (IDIN) through integrated genome-wide association studies analyzing transcription factor-driven gene networks in rat and human macrophages and monocytes [32]. A SNP associated with T1D was found to lower *Ebi2* expression and increase expression levels of IDIN genes, and ablation of *Ebi2* expression in rat macrophages increased expression of *Irf7* and IDIN genes, suggesting that *Ebi2 i*s a negative regulator of macrophage/monocyte responses [32], As IRF7 is the master regulator of IFN-α expression and pDC are the primary producers of IFN-α [12], a corollary to the association between EBI2 and type T1D would be dysregulated pDC function. However, the role of EBI2 in pDCs and IFN responses *in vivo*, have not been assessed. Here we demonstrate that EBI2 functions as a negative regulator of type I IFN responses in pDC and CD11b^+^ myeloid cells. EBI2-deficient pDCs produce higher levels of IFN-α and IFN-β in response to endosomal TLR7 and TLR9 activation. Pertussis toxin (PTX) recapitulated the EBI2-deficient phenotype in wild-type (WT) cells, suggesting that EBI2 regulates type I IFN through Gα_i_-dependent mechanisms. Dysregulated type I IFN production was corrected when EBI2 expression was restored. Additionally, EBI2 mediated pDC migration in response to 7α,25-OHC. Thus, in addition to its role as a chemotactic receptor, EBI2 regulates type I IFN production by pDCs and other myeloid cells, and allows for properly calibrated pathogenic responses.

## Materials and Methods

### Mice

*Ebi2* ^−/−^ mice were generated at Lexicon Pharmaceuticals (The Woodlands, TX) [33] and were backcrossed onto C57BL/6 background. *Ebi2* ^−/−^ and WT littermates were housed and maintained at Genentech in accordance with American Association of Laboratory Animal Care guidelines. 8–16 wk old female mice were used in all experiments. All experimental animal studies were conducted under the approval of the Institutional Animal Care and Use Committees of Genentech Lab Animal Research.

### Cell purification

Specific immune cell populations were purified from single cell suspension of splenocytes using magnetic bead separation or FACS sorting. CD4^+^ T cell, CD8^+^ T cells, B cells, pDCs and CD11b^+^ cells were purified using appropriate MACS cell isolation kits (Miltenyi Biotec). In some instances, T cells and B cells were depleted from bulk spleen cells using CD5 and CD19 MicroBeads (Miltenyi Biotec), respectively, to obtain myeloid-enriched cells. Purity of isolated cells was validated using flow cytometry and was >95% for all cell types. For isolation of myeloid cell populations by FACS, spleen cells were stained with a combination of Abs against CD11b, CD11c and mPDCA-1 (BD Biosciences). Monocyte/macrophage population was gated as CD11b^+^CD11c^−^; immature DCs were gated as CD11b^+^CD11c^int^; myeloid DCs were gated as CD11b^int^CD11c^+^; pDCs were gated as mPDCA-1^+^CD11c^int^.

### RNA isolation and quantitative RT-PCR

For analyses of *Ebi2*, *Irf7* and IDIN gene expression, pDCs were purified by magnetic bead separation using mPDCA-1 MicroBeads (Miltenyi Biotec) and CD11b^+^ cells purified using human/mouse CD11b MicroBeads (Miltenyi Biotec). RNA was isolated from purified cells using RNeasy Mini Kit (Qiagen). TaqMan real-time quantitative RT-PCR was performed using the ABI7500 Real-Time PCR system (Applied Biosystems). TaqMan Gene Expression Assay primer/probe sets were from Applied Biosystems: EBI2 (Mm02620906_s1), IRF7 (Mm00516791_g1), IFI27 (Mm01329883_gH), IFIT1 (Mm00515153_m1), IFI44 (Mm00505670_m1), MX1 (Mm00487796_m1), IP-10 (Mm00445235_m1), Ch25h (Mm00515486_s1). Expression of ribosomal protein L19 (RPL19) was used to normalize copy number. Relative expression levels (fold differences) between groups were determined by ΔΔCt method.

### Flow cytometry

Single cell suspensions were prepared from spleen, mesenteric lymph nodes, Peyer’s patches and thymus. Blood was collected by retro-orbital bleed under anesthesia. Red blood cells were lysed using ACK Lysing Buffer (Lonza). Bone marrow cells were harvested by flushing femurs with PBS. Peritoneal cells were collected by peritoneal lavage using 2–10 ml PBS. Cells were preincubated with Fc block (10% anti-FcγR, 10% normal rat serum, 10% normal mouse serum) prior to staining. Abs used for staining were all purchased from BD Biosciences: FITC-conjugated CD4, CD11c, CD21, CD23, CD38, CD44; PE-conjugated CD4, CD5, CD11b, CD43, CD69, CD138; PerCP-conjugated CD3, CD8, B220; APC-conjugated CD23, CD25, B220, mPDCA-1. Samples were acquired on a FACSCalibur flow cytometer using CellQuest Pro v5.1.1 software (BD Biosciences) and data analysis performed using FlowJo v6.4.2 software (Tree Star, Inc.). For determination of absolute cell numbers, CaliBRITE APC Beads (BD Biosciences) were used according to manufacturer’s instructions. Cell population number  =  (number of cells in specific gate/numbers of cells in live gate) x number of cells for tissue.

### In vitro cell stimulations and in vivo treatments

For *in vitro* cell stimulation experiments, pDCs and CD11b^+^ cells were purified from single cell spleen suspensions using magnetic bead separation kits (Miltenyi Biotec). 2 spleens were pooled to obtain sufficient pDC numbers. pDCs and CD11b^+^ cells were plated at 1×10^5^ cells per ml in complete RPMI (RPMI supplemented with 10% FBS, 2 mM glutamine, 2 µM 2-ME, 1 mM sodium pyruvate, 100 U/ml penicillin and 100 µg/ml streptomycin) in 96-well round-bottom plates at 200 µl final volume. Cells were stimulated with TLR9 agonist CpG-A ODN2216 (InVivoGen) at 5 µM, TLR7 agonist ssPolyU (InVivoGen) at 10 µg/ml, TLR3 agonist poly(I:C) (InVivoGen) at 50 µg/ml, or TLR4 agonist LPS (Sigma) at 1 µg/ml. For IFN-α blocking experiments, cells were preincubated with anti-IFN-α Ab at 50 µg/ml for 30 min at 37°C prior to addition of TLR agonists. All stimulations were performed for 40 hr at 37°C in a humidified incubator. For *Ebi2* expression time course studies, 2×10^5^ cells were plated in 200 µl complete RPMI in 96-well round-bottom plates and stimulated with TLR agonists as previously described. At time points ranging from 2 hr to 40 hr post-stimulation, cells were harvested for RNA isolation and real-time quantitative RT-PCR. For *Irf7* and IDIN gene expression, stimulations were performed for 24 hr, using 1×10^6^ pDCs or 2×10^6^ CD11b^+^ cells in 500 µl complete RPMI in 48-well plates.

For pDC stimulation with oxysterols, 2×10^5^ cells were plated in 200 µl complete RPMI in 96-well round-bottom plates in the presence of 7α,25-OHC (Avanti Polar Lipids, Inc.) or 25-OHC (Sigma) for 24 hr.

For PTX treatment experiments, pDC or CD11b^+^ cells were purified from 2 – 3 pooled spleens, then preincubated for 30 min with 100 ng/ml pertussis toxin from *B. pertussis* (List Biological Laboratories, Inc.) prior to stimulation.

For *in vivo* treatment with TLR ligands, mice were injected i.p. with 100 µg Imiquimod (InVivoGen) or 50 µg poly(I:C) (InVivoGen) in PBS, total volume not exceeding 300 µl. 4 hr after injection, animals were euthanized and peritoneal lavage performed and serum collected for cytokine and cellular analyses.

### LCMV infection

6 – 8 wk old female mice were infected i.v. with 2×10^6^ pfu LCMV Cl13 in 200 µl DMEM medium. Blood was collected on day 3 post-infection through retro-orbital route for determination of viral titers and type I IFN levels.

### Gene transfection

Purified pDCs were transiently transfected with EBI2 cloned into the pCMV6-AC expression vector (OriGene) or empty control vector by electroporation using Amaxa Mouse DC Nucleofector Kit, according to manufacturer’s instructions using Program Y-001. Transfected pDCs were plated at 1×10^5^ cells per ml in complete RPMI and then stimulated for 40 hr as previously described.

### ELISA

Cytokine concentrations were measured in cell culture supernatants, serum, or peritoneal lavage fluid with ELISA kits specific for IFN-α (PBL Biomedical Laboratories), IFN-β (PBL Biomedical Laboratories), TNF-α (eBioscience or R&D Systems), IL-6 (eBioscience or R&D Systems), or IL-12 (eBioscience or R&D Systems), according to manufacturer’s instructions.

### In vitro migration assay

Migration assays were performed using Costar Transwell Permeable Supports with 6.5 mm inserts and 5 µm polycarbonate membrane. Single cell splenocyte suspensions were cultured overnight in the absence or presence of 5 µM CpG-A ODN2216 or 1 µg/ml LPS, then washed and resuspended at 2×10^7^ cells/ml in complete RPMI. 100 µl of the suspension was added to a top chamber of the 24-well transwell plate. 7α,25-OHC (Avanti Polar Lipids, Inc.) or 25-OHC (Sigma) prepared in a volume of 600 µl complete RPMI were added to the bottom chamber. Cells were incubated for 3 hr at 37°C. Migrated cells in the bottom chamber were enumerated using CaliBRITE APC Beads (BD Biosciences). Nonspecific migration towards medium only was subtracted to yield specific migration towards compound in calculation of percent migrating cells. Migration index is expressed as the ratio of number of migrating cells to compound to number of migrating cells to medium only.

### Statistics

Statistical analyses were performed using JMP version 9.0.2 software (SAS Institute). We made comparisons for each pair with Student’s *t* test, with *p*-values < 0.05 considered significant.

## Results

### Exacerbated type I IFN response by Ebi2^−/−^ mice upon TLR ligand challenge or viral infection

**Figure 1:**
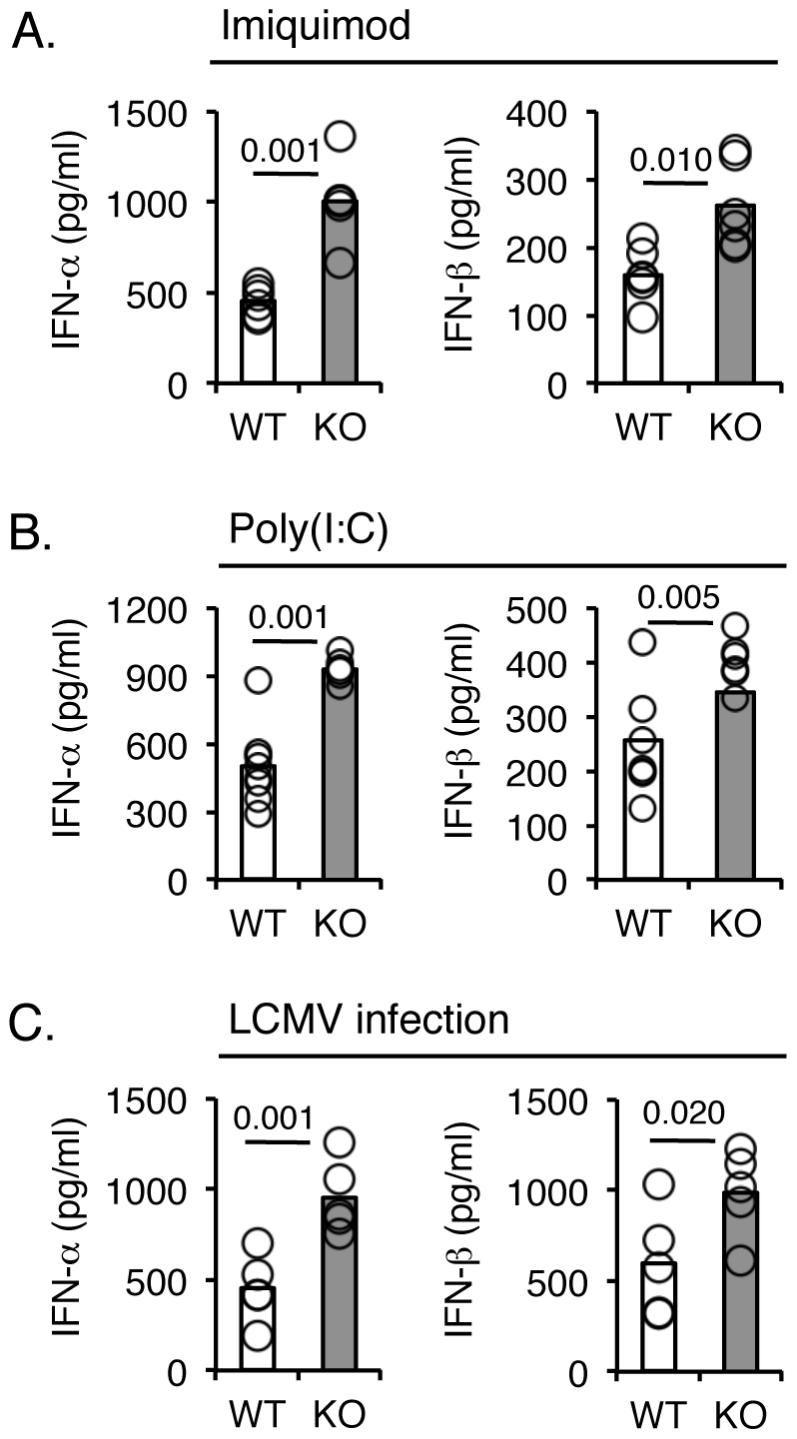
*Ebi2* ^−**/**−^ mice have stronger pDC-mediαted type I IFN responses to *in vivo* challenge with TLR agonists or LCMV Cl13. **A, B.** Type I IFN production in mice challenged i.p. with TLR7 agonist Imiquimod (**A**) or TLR3 agonist poly(I:C) (**B**). IFN-α and IFN-β concentrations in peritoneal lavage fluid 4 hr following challenge were determined by ELISA. Bars represent mean values (white denotes WT, shaded denotes KO; n = 5 per group); circles represent individual animals. **C**. LCMV Cl13 infection in *Ebi2* ^−/−^ mice. *Ebi2* ^−/−^ (shaded bars) or WT littermate (white bars) mice were infected with 2×10^6^ PFU LCMV Cl13 i.v. On day 3, serum type I IFN concentrations were determined by ELISA. Bars denote mean values; circles represent individual animals (n = 5 per group). *P*-values are denoted when considered statistically significant (*p*<0.05). IFN-α concentrations in naïve mice were below assay limit of detection.

Since EBI2 has been genetically identified as a possible regulator of a network of inflammatory genes known to be regulated by IRF7 [32], we asked if EBI2 regulates type I IFN responses. *Ebi2* ^−/−^ and WT littermate mice were challenged with intraperitoneal injections of the TLR7 agonist imiquimod. Higher concentrations of IFN-α and IFN-β were measured in the peritoneal lavage fluid of *Ebi2* ^−/−^ mice compared to WT littermates (Figure 1A; 2.23-fold higher for IFN-α, 1.63-fold higher for IFN-β; *p* = 0.001, 0.010, respectively). Similarly, TLR3-induced type I IFN were also elevated in *Ebi2* ^−/−^ mice compared to WT littermates (Figure 1B; 1.85-fold higher for IFN-α, 1.34-fold higher for IFN-β; *p* = 0.001, 0.005, respectively). These results indicated that EBI2 may directly regulate type 1 IFN responses, independent of MyD88, a key mediator of TLR7 but not TLR3 signaling [34]. To test the physiological relevance of EBI2 in TLR-mediated type 1 IFN production, we infected *Ebi2* ^−/−^ and WT littermate mice with lymphocytic choriomeningitis virus (LCMV) clone 13. In this model of viral infection, type 1 IFN production is rapidly induced via a TLR7–dependent process. Peripheral type 1 IFN levels were higher in *Ebi2* ^−/−^ mice infected with virus 3 days post-infection compared to WT littermates, (Figure 1C; 2.12-fold higher for IFN-α, 1.66-fold higher for IFN-β; *p* = 0.001, 0.020, respectively). Taken together, these data demonstrated an important role for EBI2 in limiting TLR-mediated type 1 IFN production *in vivo*.

### EBI2 is expressed by plasmacytoid and myeloid dendritic cells

**Figure 2:**
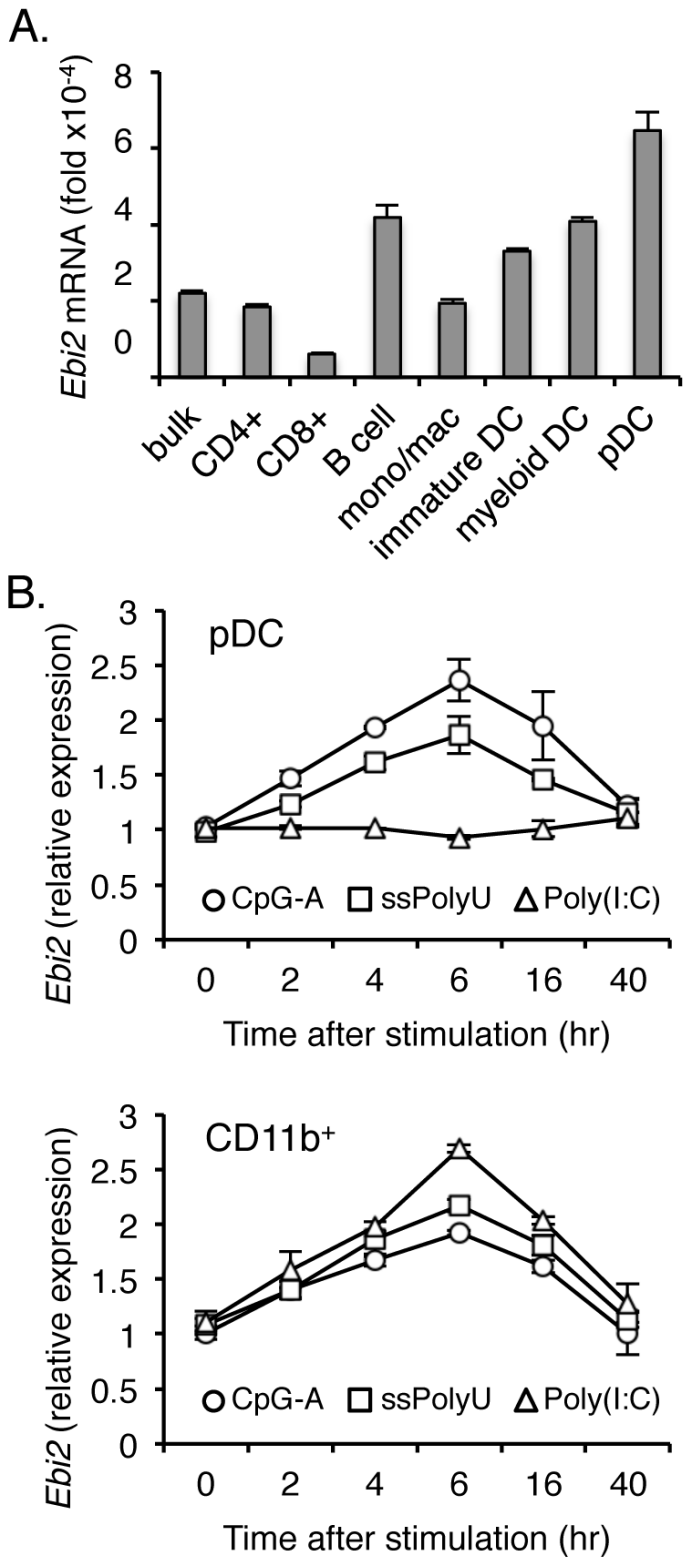
Immune cell expression of EBI2. **A**. Quantitative PCR analysis of *Ebi2* transcript abundance was performed in purified T cells, B cells and indicated myeloid cell populations. **B**. Expression of *Ebi2* in TLR-stimulated pDCs and CD11b^+^ myeloid cells. *Ebi2* expression in purified pDCs and CD11b^+^ myeloid cells that were stimulated for various time points over a 40 hr period with indicated TLR agonist. *Ebi2* expression in each cell type was normalized to *RPL19* expression, and data are shown as *Ebi2* expression relative to unstimulated cells at the corresponding time point. Data are shown as mean ± s.d. of two independent experiments.

The cellular sources of type I IFN vary depending on the type of challenge or infection, but in general, pDCs and myeloid cells are the predominant cell types triggered by activation of endosomal TLR [35]. Therefore, we assessed the role of EBI2 directly in these cells. Consistent with human myeloid DCs [26], [28], resting mouse splenic myeloid DCs expressed constitutive levels of *Ebi2* similar to those found in B cells (Figure 2A). In addition, splenic pDCs also expressed *Ebi2* at comparable levels. Upon activation with endosomal TLR agonists to TLR7 and TLR9, *Ebi2* expression peaked 6 hr post-stimulation in both pDCs and CD11b^+^ cells (2.37-fold and 1.87-fold increase for pDC, 1.92-fold and 2.17-fold increase for CD11b^+^ cells, respectively), with expression remaining elevated after 16 hours (1.95-fold and 1.46-fold increase for pDC. 1.62-fold and 1.81-fold increase for CD11b^+^ cells, respectively) and returning to constitutive levels by 40 hours (Figure 2B). pDCs do not express TLR3 and, as expected, *Ebi2* expression in pDCs was unaffected by stimulation with Poly(I:C) (Figure 2B). This pattern of *Ebi2* expression in pDC and CD11b^+^ myeloid cells is consistent with surface expression of EBI2 in DC subsets reported by others [28]. The constitutive expression of *Ebi2,* and its transient increased expression after activation, suggests it may function as a tonic regulator of immune responses.

### EBI2 ligand 7α,25-OHC is a chemoattractant for pDCs

**Figure 3:**
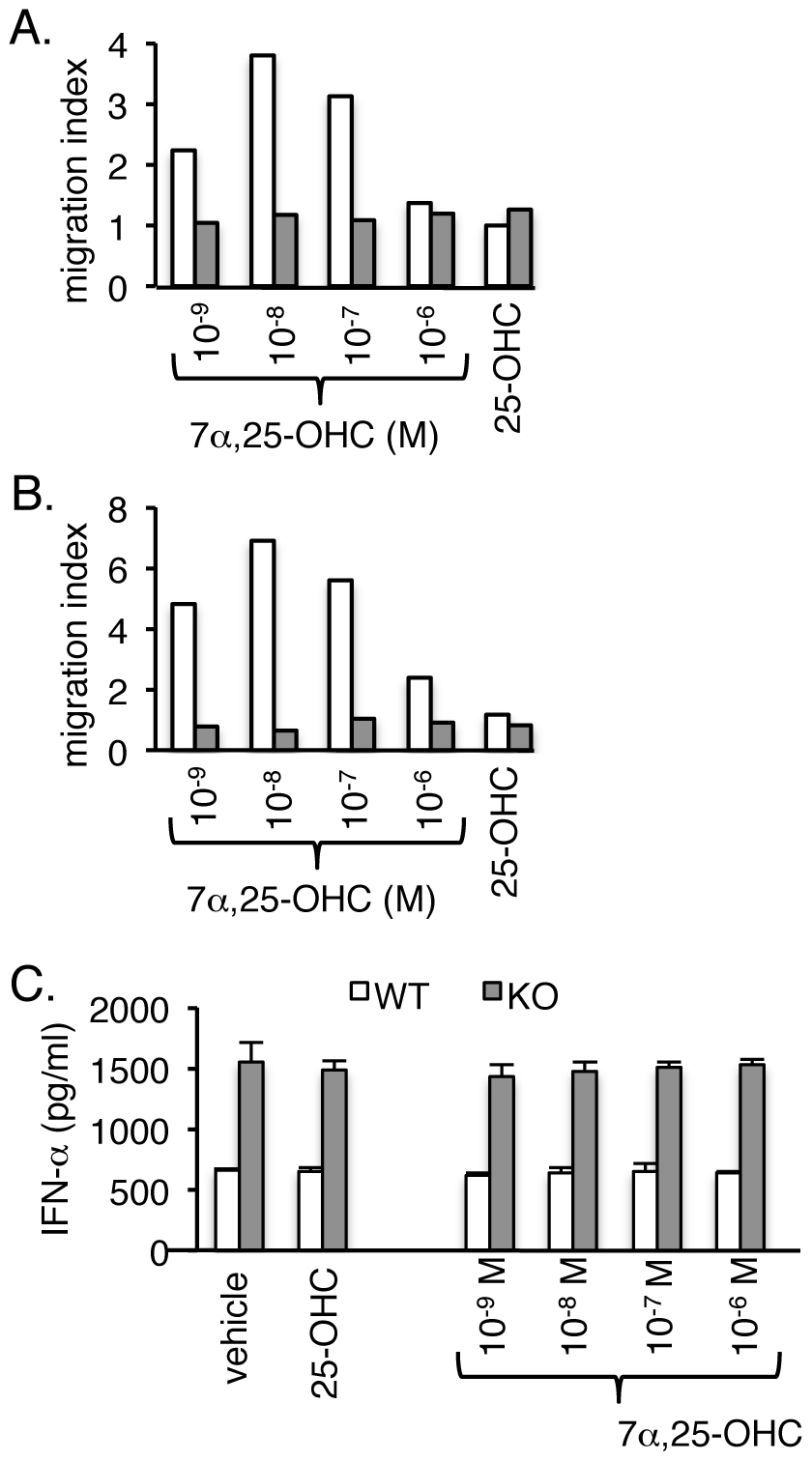
EBI2 expressed by type I IFN-producing cells functions as a chemotactic receptor. **A**, **B**. EBI2 ligand 7α,25-OHC induces migration of pDCs. *In vitro* migration studies of unstimulated (**A**) or TLR9 agonist CpG-A ODN2216 activated (**B**) pDCs from EBI2 WT (white bars) or KO (shaded bars) mice towards 7α,25-OHC (indicated concentrations) or 25-OHC (10^−6^ M). Starting cells and migrating cells were analyzed by specific staining for pDCs and flow cytometry. Data shown are representative of two separate experiments. **C**. Stimulation of activated pDCs with 7α,25-OHC does not affect IFN-α production. pDCs activated with CpG-A were stimulated for 40 hr with 25-OHC (10^−6^ M) or 7α,25-OHC. IFN-α concentrations in cell supernatants were determined by ELISA. Data are shown as mean ± s.d. from a single experiment with duplicate measurements. The experiment was repeated with similar results.

The ligand for EBI2 is oxysterol 7α,25-OHC [26], [27], acts as a chemotattractant for B cells, T cells, DCs (particularly splenic CD4^+^ DCs), and to a lesser extent macrophages and natural killer cells [23], [27]–[29]. Given that pDC constitutively express EBI2, we hypothesized that 7α,25-OHC would also affect pDC migration. To test this hypothesis, we cultured splenocytes from WT and EBI2-deficient mice with 7α,25-OHC in a transwell system. 7α,25-OHC mediated a dose-dependent migration response in WT cells but not EBI2-deficient cells (Figure S1A). Analysis of specific pDC migration showed that WT pDCs were responsive to 7α,25-OHC (Figure 3A), and specific B cell migration was similar to previously published reports (Figure S1D) [26], [27]. When cells were prestimulated with TLR9 agonist CpG-A ODN2216, greater migration of the bulk splenocyte population towards 7α,25-OHC was observed (Figure S1B), which may be a consequence of more robust pDC migration (Figure 3B) and the increased EBI2 expression in pDCs following CpG-A stimulation (Figure 2B). B cell migration was not affected by TLR9 stimulation (Figure S1E), but was enhanced with LPS stimulation (Figure S1C, F). As expected, EBI2-deficient cells, specifically pDCs and B cells, were not responsive to 7α,25-OHC (Figure 3A, B, Figure S1). Consistent with previous reports, the 7α,25-OHC activity curve was bell-shaped, indicating reduced attraction at high ligand concentrations that may be due to ligand-induced receptor desensitization or internalization [26], [27]. Additionally, 25-OHC, the precursor to 7α,25-OHC, failed to stimulate migration of pDCs, confirming the specificity of EBI2 for 7α,25-OHC (Figure 3A, B).

To test whether 7α,25-OHC could activate pDCs directly, naïve pDCs were stimulated with 7α,25-OHC and type I IFN production measured. At a dose range from 10^−9^ to 10^−6^ M, 7α,25-OHC did not measurably stimulate pDCs (data not shown). Moreover, after activation of pDC with CpG-A, 7α,25-OHC did not alter IFN-α production by WT or EBI2-deficient pDCs (Figure 3C). Collectively, these data indicated that EBI2 functions as a chemotactic receptor in pDCs in a similar manner to its function in other immune cell types.

### EBI2^−/−^ mice have fewer pDC and myeloid DC in spleen and peritoneal cavity

**Figure 4:**
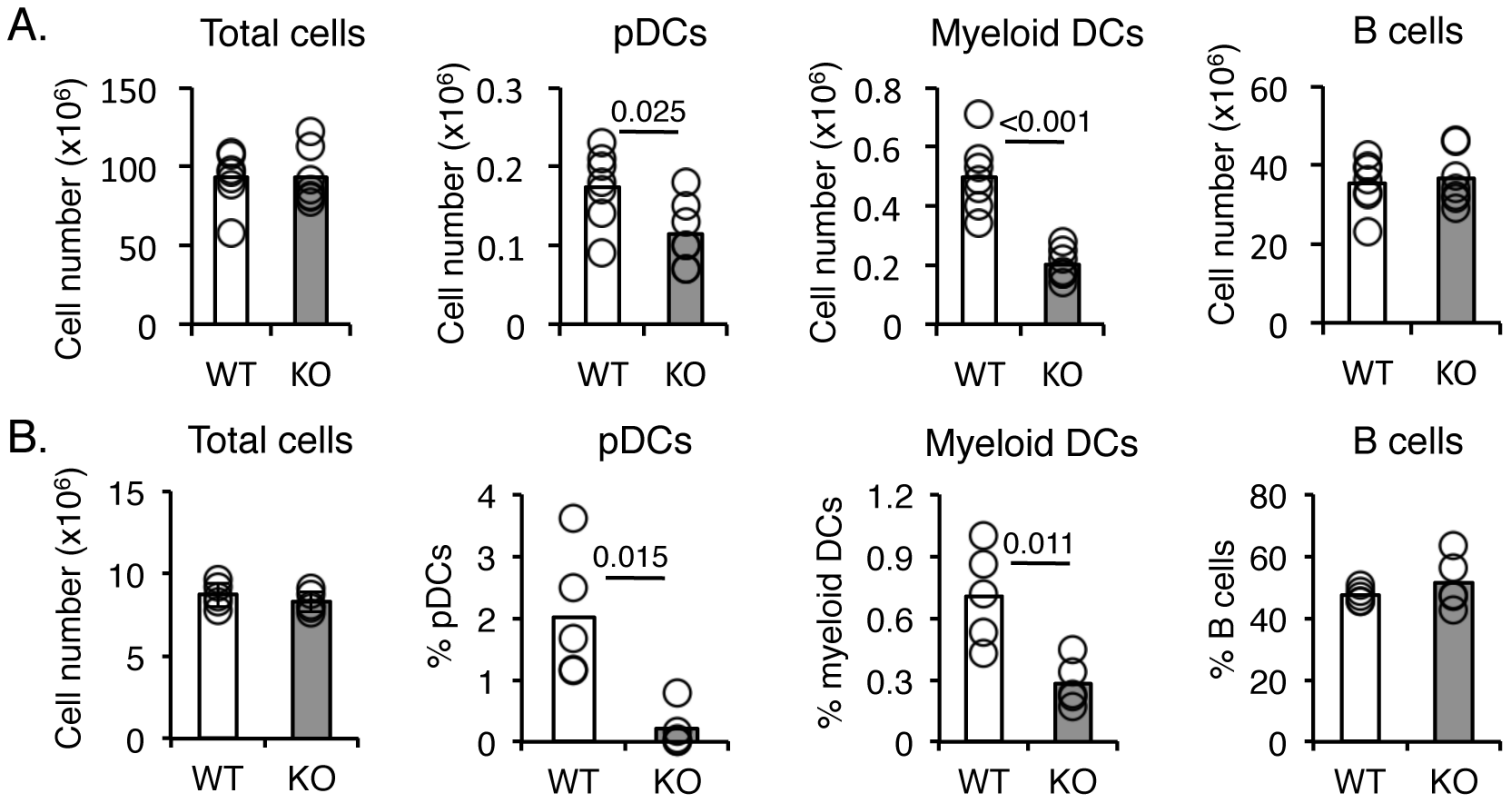
Immune cell distribution in *Ebi2* ^−**/**−^ mice. Immune cell distribution in spleen (A) or peritoneal lavage fluid (B) of naïve WT and *Ebi2* ^−/−^ mice. Flow cytometric analysis was used to enumerate pDCs, myeloid DCs and B cells in WT (white bars) or *Ebi2* ^−/−^ (shaded bars) mice (n = 7 per group for spleens; n = 5 per group for peritoneal lavage fluid). Bars represent mean values; white denotes WT, shaded denotes KO; circles represent individual animals. *P*-values are denoted when considered statistically significant (*p*<0.05).

We then assessed whether the EBI2/7α,25-OHC pathway was required for pDC trafficking *in vivo*. In naïve animals, numbers of pDCs in EBI2-deficient spleens were reduced by approximately 35% and myeloid DCs by nearly 60% (Figure 4A; *p* = 0.025 for pDCs, *p*<0.001 for myeloid DCs). Deficiencies were also observed in the peritoneum, with the frequency of pDCs and myeloid cells in the peritoneal lavage fluid reduced by approximately 90% and 60% in *Ebi2* ^−/−^mice compared to WT littermates, respectively (Figure 4B; *p* = 0.015 fro pDCs, *p* = 0.011 for myeloid DCs). Cell frequencies were normal in peripheral blood (Table S1). No differences in overall total cell numbers in either spleen or peritoneal lavage fluid were observed between *Ebi2* ^−/−^ and WT littermates (Figure 4), suggesting that the decrease in EBI2^−/−^ mice was specific to pDCs and myeloid DCs. The reduction in myeloid DCs was consistent with previously published data showing a reduction in total splenic DCs in *Ebi2* ^−/−^ mice [28], [29]. Immunophenotyping of other cell types in the spleen of *Ebi2* ^−/−^ mice showed that all B and T cell populations were found in normal numbers and frequencies (Figure S2, Table S1), consistent with previously reports [24], [25].

### EBI2-deficient pDC have elevated IFN-α responses to TLR stimulation

**Figure 5:**
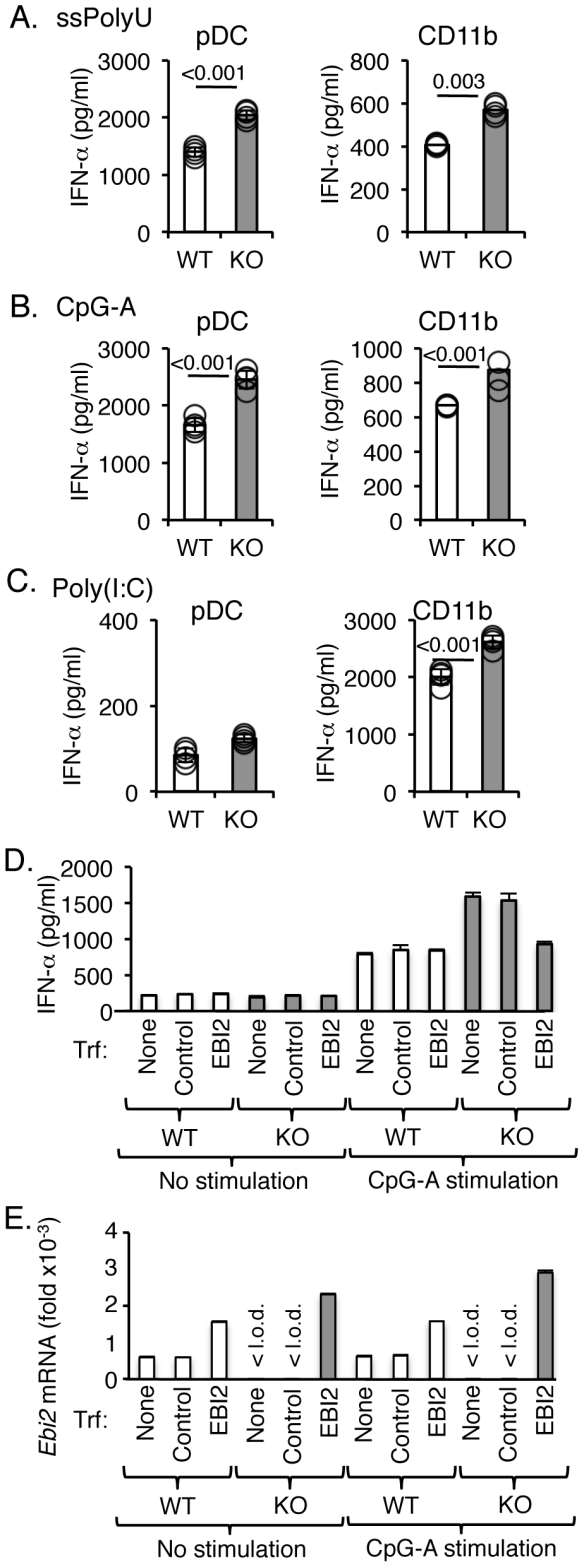
pDCs from *Ebi2* ^−/−^ mice produce more IFN-α when activated with TLR7 or TLR9 agonists. pDCs or CD11b^+^ monocyte/macrophages from *Ebi2* ^−/−^ or WT littermate mice were stimulated with TLR7 agonist ssPolyU (**A**), TLR9 agonist CpG-A ODN2216 (**B**) or TLR3 agonist poly(I:C) (**C**). IFN-α secretion in culture supernatants after 40 hr stimulation were measured by ELISA. Cells were purified from pools of 2 spleens each. 8 mice from each group were used, resulting in 4 pools. Bars represent mean values (white denotes WT, shaded denotes KO); circles represent each pool. *P*-values are denoted when considered statistically significant (*p*<0.05). IFN-α was not detectable in the absence of stimulation. **D**. Rescue of EBI2 expression in EBI2-deficient pDCs reduces type I IFN response to normal levels. EBI2 KO (shaded bars) or WT (white bars) pDCs were transiently transfected with EBI2 or control vector, counted and plated, then stimulated with TLR9 agonist CpG-A ODN2216. IFN-α concentrations in supernatants were measured by ELISA 40 hr after stimulation. Data are shown as mean ± s.d. of duplicate measurements. The experiment was repeated twice with similar results in each. **E**. Quantitative PCR analysis of *Ebi2* transcript abundance was performed with *Ebi2* expression presented relative to *RPL19* expression. Data are shown as mean ± s.d. of triplicate measurements; < l.o.d. denotes below limit of detection.

The chemotactic function of EBI2 in pDCs and myeloid DCs raised a key question: is the exacerbation of IRF7-mediated type 1 IFN responses observed in EBI2-deficient mice *in vivo* a byproduct of dysregulated pDC trafficking or is it the result of a direct, cell-intrinsic role for EBI2 in IRF7 signaling? To address this issue, we determined if purified *Ebi2* ^−/−^ pDCs were hyperresponsive to TLR stimulation *in vitro*. When activated with the TLR7-agonist ssPolyU or the TLR9-agonist CpG-A ODN2216, purified splenic pDCs from *Ebi2* ^−/−^ mice expressed a significant 50% elevation in levels of IFN-α as compared to WT pDCs (Figure 5A, B; *p*<0.001 under both conditions). TLR7 and TLR9 signaling also elicited enhanced production of pro-inflammatory cytokines TNF-α, IL-6 and IL-12 by EBI2-deficient pDCs, but this could be attributed to secondary signaling through IFNαR as anti-IFN-α mAb abrogated the increased pro-inflammatory cytokine production, resulting in EBI2-deficient and WT pDCs producing similar amounts of TNF-α, IL-6 and IL-12 (Figure S3). Similarly, activation of purified splenic CD11b^+^ cells with the TLR7 or TLR9 agonists induced 30 to 40% higher levels of IFN-α from EBI2-deficient myeloid cells than WT cells (Figure 5A, B; *p* = 0.003 for ssPolyU, *p*<0.001 for CpG-A;). As observed with pDCs, pro-inflammatory cytokine production by EBI2-deficient myeloid DCs was similar to WT in the presence of anti-IFN-α mAb (Figure S3). TLR3 stimulation also elicited a 30% increase in IFN-α levels from EBI2-deficient CD11b^+^ cells (Figure 5C; *p*<0.001,). As expected, a minimal IFN-α response was measured from pDCs since pDCs lack TLR3 expression.

To confirm the increased levels of Type 1 IFNs in EBI2^−/−^ cells were specific to EBI2, the knockout cells were reconstituted with EBI2 and TLR-mediated cytokine production assessed. Wild-type levels of IFNs were fully restored in *Ebi2* ^−/−^ cells transfected with EBI2-expressing vectors. Overexpression of EBI2 in *Ebi2* ^−/−^ pDC decreased IFN-α production in response to CpG-A stimulation, compared to untransfected or control vector-transfected EBI2-deficient pDCs, and levels were comparable to activated WT pDCs (Figure 5D). Overexpression of EBI2 in WT pDCs did not have an effect, as untransfected and nucleofected WT pDCs produced similar levels of IFN-α. CpG-A stimulation did not have an effect on *Ebi2* expression levels in either WT pDCs or transfected EBI2-deficient pDCs (Figure 5E). Thus, cell expression of EBI2 directly impacts elaboration of IFN-α via the MyD88-IRF7 pathway, and negatively regulates type I IFN production.

### Pertussis toxin treatment phenocopies EBI2 deficiency

**Figure 6:**
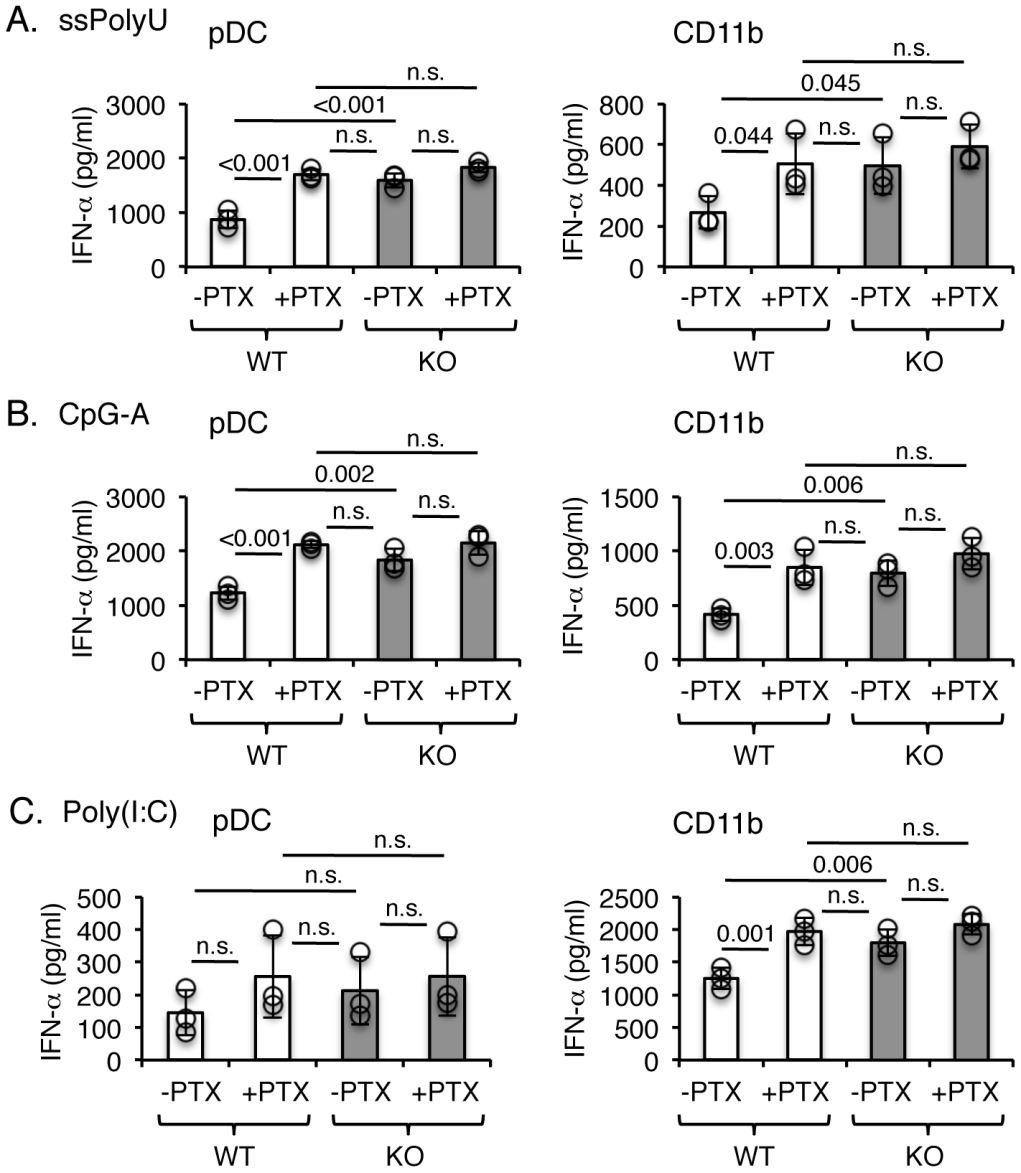
Type I IFN production is enhanced by pertussis toxin treatment. pDCs or CD11b^+^ monocyte/macrophages from WT or *Ebi2* ^−/−^ littermate mice were preincubated with 100 ng/ml PTX, and then stimulated with TLR7 agonist ssPolyU (**A**), TLR9 agonist CpG-A ODN2216 (**B**) or TLR3 agonist poly(I:C) (**C**). IFN-α secretion in culture supernatants after 40 hr stimulation were measured by ELISA. Bars represent mean values of 3 independently performed experiments (white denotes WT, shaded denotes KO); circles represent individual experiments. *P*-values are denoted when considered statistically significant (*p*<0.05); n.s. denotes not statistically significant. IFN-α was not detectable in the absence of stimulation.

Because EBI2 signaling is coupled to Gα_I_ [23], we asked whether the EBI2-mediated dampening of type I IFN responses required G protein-regulated pathways. PTX mediates ADP-ribosylation of Gα_i_, leading to inactivation of signaling, thereby allowing assessment of proximal signaling. Pretreatment of WT pDCs and CD11b^+^ cells with PTX resulted in significantly higher amounts of IFN-α in response to stimulation with endosomal TLR agonists (Figure 6). The levels of IFN-α produced by WT cells following PTX treatment were similar to untreated EBI2-deficient cells, and no differences were observed between WT and KO cells treated with PTX. These data suggest that EBI2 regulation of type I IFN responses is dependent on Gα_i_-controlled pathways, and that EBI2 may proximally modulate expression of IRF7 and/or the IRF7-driven inflammatory gene network.

### EBI2 negatively regulates IRF7-dependent pathways in pDC

**Figure 7:**
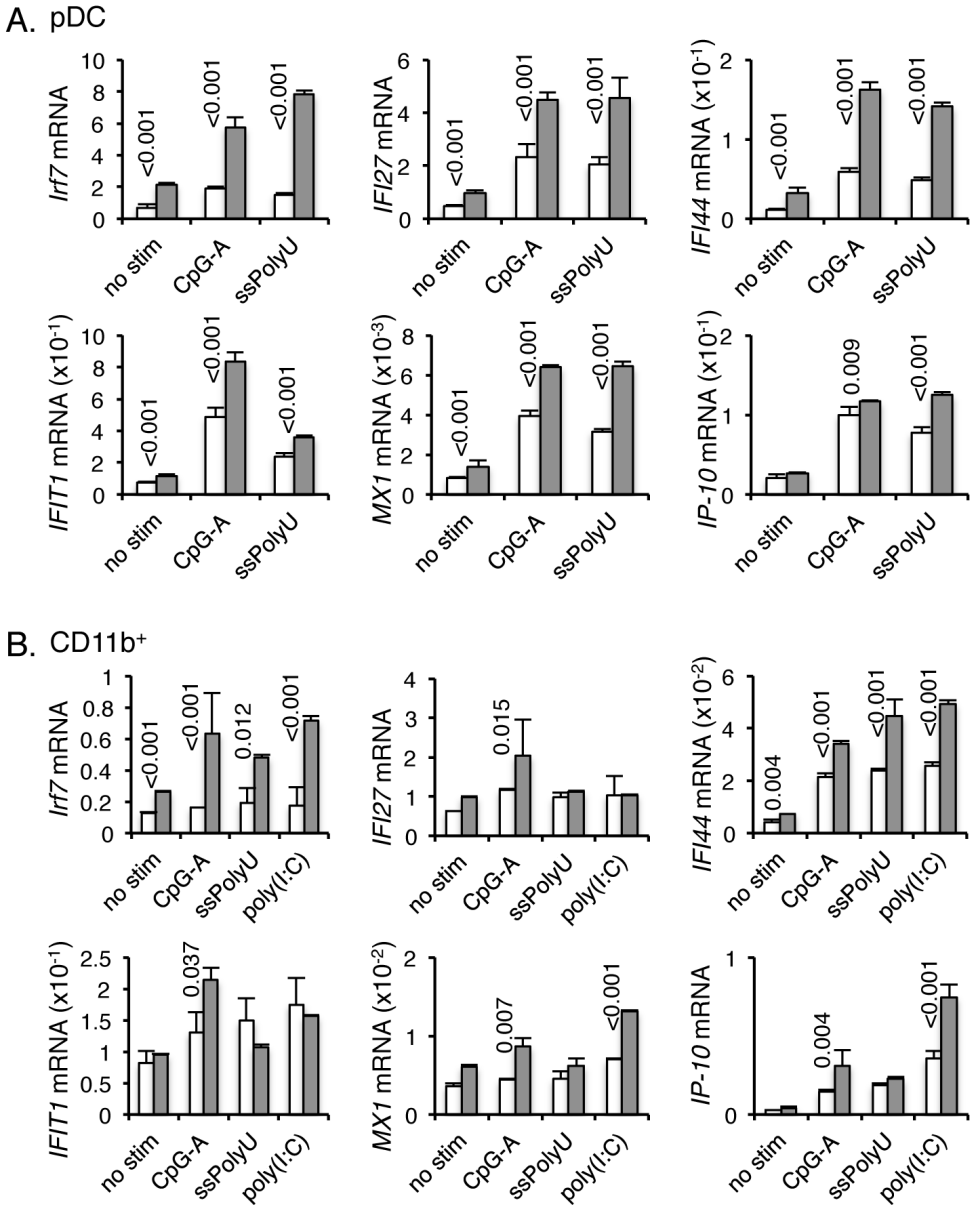
Expression of IRF7 and IDIN genes in TLR-stimulated EBI2-deficient pDCs and monocytes/macrophages. IDIN gene expression was determined by real-time RT-PCR. Expression in unstimulated or TLR9 agonist CpG-A ODN2216, TLR7 agonist ssPolyU, TLR3 agonist poly(I:C) or TLR4 agonist LPS stimulated pDCs (**A**) or CD11b^+^ monocytes/macrophages (**B**) from WT (white bars) or EBI2 KO (shaded bars) mice. Gene expression is presented relative to *RPL19* expression. Data are shown as mean ± s.d. from a single experiment with duplicate measurements. The experiment was repeated with similar results.

We next directly assessed the role of EBI2 on *Irf7* expression and IDIN downstream genes in pDC and CD11b^+^ myeloid cells. In naïve pDCs purified from spleen, transcripts for *Irf7* were at least 2-fold higher in EBI2-deficient cells compared to WT cells (Figure 7A). In CD11b^+^ cells, *Irf7* was also more highly expressed in EBI2-deficient cells (Figure 7B). Expression levels of IDIN genes *Ifi27*, *Ifi44*, *Ifit1*, *Mx1* and *Ip-10* were notably higher in naïve pDCs isolated from EBI2 KO spleens compared to WT (Figure 7A). Naïve EBI2-deficient CD11b^+^ cells also had higher expression of all measured IDIN genes, with the exception of *Ifit1*, but the magnitudes of increased expression were lower than observed in pDCs (Figure 7B). When activated with TLR9 or TLR7 agonists, IDIN gene expression was upregulated in WT and EBI2-deficient pDCs (Figure 7A). The increased expression observed in EBI2-deficient pDCs reflected the increased initial constitutive expression in EBI2-deficient pDCs relative to WT pDCs, as the fold-increase in gene expression following stimulation compared to naïve, unstimulated cells was similar for both EBI2-deficient and WT pDCs. While *Irf7* expression increased by at least 2-fold upon stimulation with CpG-A and ssPolyU, increases in IDIN gene expression were generally more dramatic (Figure 7A). For CD11b^+^ cells, changes in *Irf7* expression were not as great as observed for pDCs (Figure 7B). As with pDCs, the magnitude of changes in IRF7 and IDIN gene expression between EBI2-deficient and WT CD11b^+^ cells was comparable relative to unstimulated cells. Unlike pDCs where there was a general increase in expression of all IDIN genes examined, only *Ifi44* and *Ip-10* were exceptionally elevated in CD11b^+^ cells following stimulation with various TLR ligands (Figure 7B). These data demonstrate that EBI2 negatively regulates basal expression of IRF7 and IDIN genes, dampening type 1 IFN production upon IRF7-mediated stimulation.

## Discussion

Our findings define a role for EBI2 as a direct negative regulator of type I IFN responses, and its constitutive surface expression on pDC and myeloid cells and further transient increased expression upon activation with TLR stimulation help to regulate against aberrant type I IFN expression. EBI2-deficient pDCs and mDC produced higher levels of IFN-α and IFN-β in response to endosomal TLR activation. This dysregulated type I IFN production was abrogated when EBI2 expression was rescued, resulting in restoration of normal pDC responses. EBI2-deficient pDCs expressed higher basal transcript levels of *Irf7* and IDIN genes, suggesting that knockout pDCs are more primed to produce type I IFNs upon stimulation than their WT counterparts. The elevated *in vitro* response of EBI2-deficient pDCs to TLR stimulation was recapitulated when *Ebi2* ^−/−^ mice were challenged with TLR ligands or infected with LCMV. pDCs are also responsive to EBI2 ligand 7α,25-OHC-stimulated migration, and naïve *Ebi2* ^−/−^ mice were found to have fewer numbers of pDCs and myeloid DCs in the spleen and lower frequencies of these cell types in the peritoneum when compared to WT mice – a surprising finding given the enhanced type 1 IFN responses mounted by these animals.

Type 1 IFNs are a group of pleiotropic cytokines that are important for protection against viral infections, however, dysregulated type I IFN responses may precipitate and perpetuate autoimmune diseases [1]. A key regulator of the type I IFN response is IRF7 [12], and high basal expression of IRF7 by pDCs contributes to their ability to rapidly secrete IFNs. Aberrant expression of type I IFNs underscores the pathogenesis of a number of autoimmune diseases driven largely by host-derived nucleic acids. An IFN-regulated gene signature is particularly prominent in SLE and also found in T1D, RA, MS, psoriasis and Sjogren’s syndrome [14]–[22], [36]. EBI2 was demonstrated to moderate type I IFN responses, particularly by pDCs. In the absence of EBI2, pDCs had higher IRF7 expression and elaborated elevated type I IFN responses upon stimulation, suggesting that EBI2 plays an inhibitory role to dampen type I IFN production. Furthermore, we show that EBI2 is a regulator of IRF7, a key driver of this inflammatory network [32]. EBI2-deficient mice had higher constitutive expression of IRF7 as well as IDIN genes. Previously, a single nucleotide polymorphism associated with T1D risk was identified as lowering EBI2 expression and increasing expression of IDIN genes. This IDIN defined in TID is similar to IFN signatures described in other immune diseases such as SLE and RA [16], [18].

EBI2 has been described as having constitutive activity through Gα_I_ [23], and interaction with its ligand, 7α,25-OHC, induces GTP-γS binding, suppression of cAMP accumulation, and release of intracellular calcium [26], [27]. Gα_i_ is one subunit of the heterotrimeric G protein, which also contains the Gβγ complex. Upon ligand interaction with GPCR, Gα_i_ detaches from Gβγ to activate downstream effector pathways [37], [38]. It has been reported that in the absence of Gα_i2,_ activated DCs exhibit profoundly higher IL-12, TNF-α and IL-23 production [39], [40]. PTX mediates ADP-ribosylation of Gα_i_, leading to inactivation of signaling, thereby allowing assessment of whether proximal signaling events facilitated by Gα_i_ are required. We show that pretreatment of WT pDCs and CD11b^+^ cells with PTX resulted in significantly higher amounts of IFN-α in response to stimulation with various endosomal TLR agonists, and that these levels of IFN-α were similar to untreated EBI2-deficient cells. These data suggest that EBI2 regulation of type I IFN responses is dependent on Gα_i_-controlled pathways, and that EBI2 may proximally modulate expression of IRF7 and/or the IRF7-driven inflammatory gene network.

pDCs express various surface receptors that, when activated, inhibit type I IFNs and proinflammatory cytokine and chemokine production, including ILT7, BDCA2 and Siglec-H. These surface molecules require additional interactions with immunoreceptor tyrosine-based activation motif (ITAM)-containing receptors to provide signals to shut down IFNs; both BDCA2 and ILT7 associate with the FcεRIγ adaptor [41]-[44]; whereas Siglec-H associates with the adaptor protein, DAP12 [45]. It is unlikely that EBI2 associates with an ITAM adaptor, as there is no reported evidence for phosphorylation of Src or Syk family kinases, hallmarks of ITAM-mediated signaling, following EBI2 activation. While the specific mechanism(s) by which EBI2 regulates type I IFN responses remains unknown, the constitutive activity of EBI2 and its transient increased expression upon TLR stimulation suggests EBI2 provides a check against aberrant type I IFN expression.

In addition to directly regulating pDC type I IFN responses through IRF7, EBI2 may also influence pDC responses by guiding their migration to and localization within lymphoid tissues or, in the case of autoimmune diseases, target organs. pDCs are produced in the bone marrow and then migrate to LN, spleen and mucosal-associated lymphoid tissues under steady-state conditions [5], [46], [47]. In spleens of naïve mice, pDCs are found scattered in the T cell area and in the red pulp, with cells rarely detected in the marginal zone [48], [49]. After treatment with TLR7 or TLR9 agonists, most splenic pDCs are found in clusters within the marginal zone and in the T cell area, with some isolated pDCs still detected in the red pulp [49]. As some autoimmune diseases are characterized by aberrant frequencies of circulating pDCs and accumulation of pDCs in target organs or tissues [47], [50], [51], it will be interesting to determine whether the EBI2-7α,25-OHC axis plays a role in pDC migration/localization and activation of pathogenic pDC in tissues.

Our findings identify a novel role for EBI2 in regulation of type I IFN responses by pDCs and myeloid DCs. While our studies focused primarily on the MyD88-IRF7-dependent pathway, our data suggest that the negative regulatory role of EBI2 may apply to MyD88-independent pathways, such as IRF3, as well. The PTX sensitivity of pDCs and myeloid DCs strongly suggests that EBI2, through its coupling with Gα_i_, is a proximal regulator of a broad number of pathways influencing type I IFN responses. The challenge for future studies is to further define the mechanism(s) by which EBI2 exerts regulatory control of type I IFN responses in the face of balancing aversion to self-nucleic acids with a state of readiness against viral pathogens. Our data provide insight into mechanisms by which EBI2 deficiency leads to dysregulated type I IFN production, a key driver of many autoimmune diseases, and reveals the EBI2/7α,25-OHC axis as a potential novel pathway in autoimmune disease pathogenesis.α.

## Supporting Information

Figure S1 **EBI2 ligand 7α,25-OHC induces cell migration.** *In vitro* migration studies of unstimulated (**A**, **D**), TLR9 agonist CpG-A ODN2216 activated (**B**, **E**) or LPS stimulated (**C**, **F**) bulk splenocytes (**A**-**C**) or B cells (**D**-**F**) from EBI2 WT (white bars) or KO (shaded bars) mice towards 7α,25-OHC (indicated concentrations) or 25-OHC (10^−6^ M). Starting cells and migrating cells were analyzed by flow cytometry. B cell migration was determined by specific staining in the bulk splenocyte population. Data shown are representative of two separate experiments.
(TIF)

Figure S2 **CD4^+^ and CD8^+^ T cell distribution in** ***Ebi2*** ^−**/**−^ **and wild-type mice.** **A**. T cell distribution in spleen of naïve WT and *Ebi2* ^−/−^ mice. Flow cytometric analysis was used to enumerate CD4^+^ and CD8^+^ T cells in spleens of WT (white bars) or *Ebi2* ^−/−^ (shaded bars) mice (n = 7 per group). **B**. T cell frequencies in peritoneal lavage fluid of naïve WT and *Ebi2* ^−/−^ mice (n = 5 per group). **C**. T cell distribution in peritoneal lavage fluid of Imiquimod-challenged mice 4 hr after i.p. injection (n = 6 per group). Bars represent mean values; white denotes WT, shaded denotes KO; circles represent individual animals.
(TIF)

Figure S3 **Elevated pro-inflammatory cytokine responses by EBI2-deficient pDCs and monocytes/macrophages activated with TLR7 or TLR9 agonists are due to IFN-α-mediated secondary signaling.** pDCs and CD11b^+^ cells from *Ebi2* ^−/−^ or WT littermate mice were pre-incubated in the absence or presence of anti-IFN-α mAb, then stimulated with TLR7 agonist ssPolyU (**A**) or TLR9 agonist CpG-A ODN2216 (**B**). TNF-α IL-6 and IL-12 in culture supernatants after 40 hr stimulation were measured by ELISA. Cells were purified from pools of 2 spleens each. 8 mice from each group were used, resulting in 4 pools. Bars represent mean values (white denotes WT, shaded denotes KO); circles represent each pool. *P*-values are denoted when considered statistically significant (*p*<0.05).
(TIF)

Table S1 **Immune cell populations from various tissues in EBI2-deficient mice.**
(DOC)
