# Supplementary figures and images for: EBI2 Is a Negative Regulator of Type I Interferons in Plasmacytoid and Myeloid Dendritic Cells
Source: PLoS One. 2013 Dec 26;8(12):e83457. doi: 10.1371/journal.pone.0083457 (PMC3873289; doi:10.1371/journal.pone.0083457)

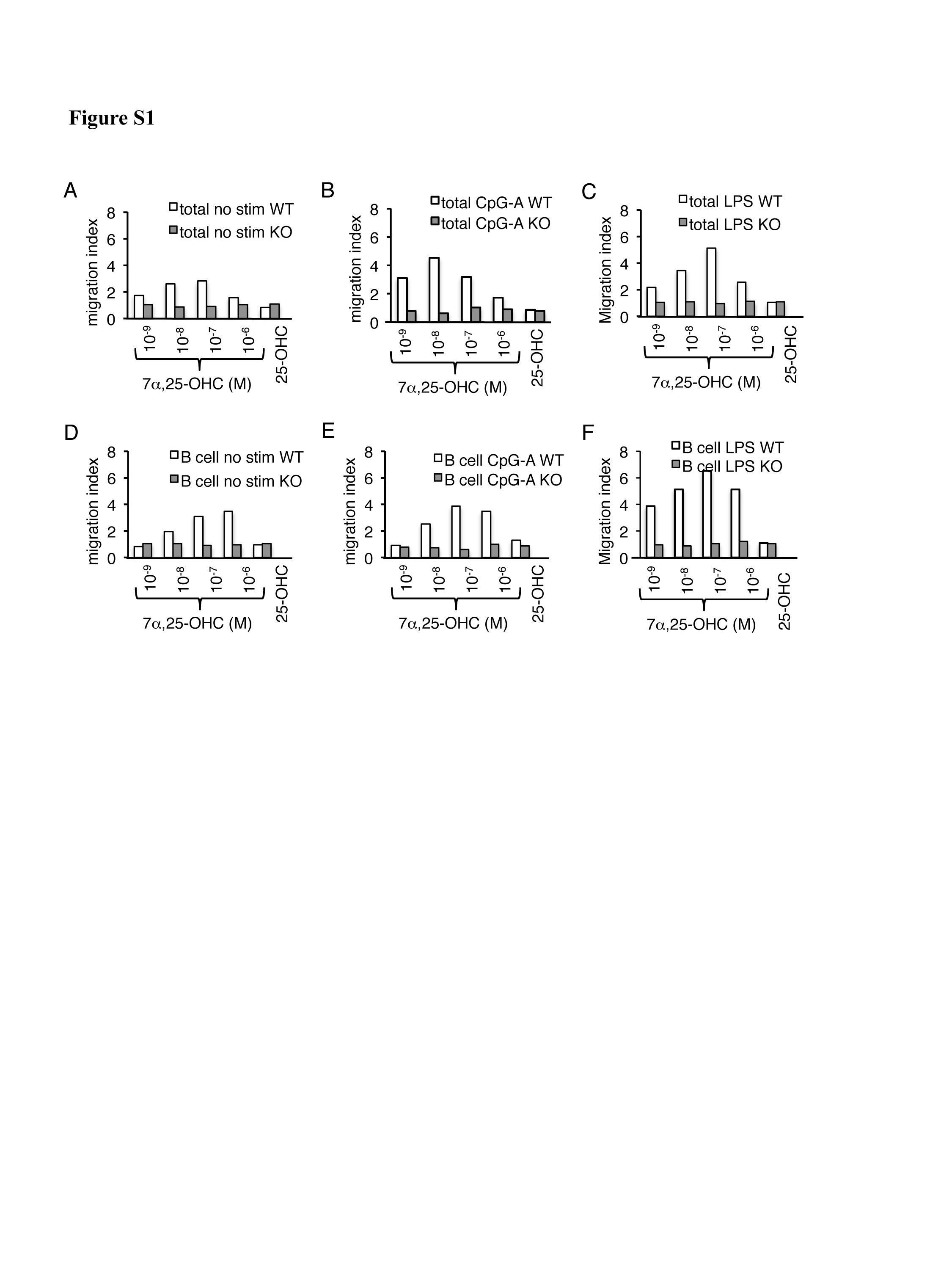

Supplement: Figure S1 — EBI2 ligand 7α,25-OHC induces cell migration. In vitro migration studies of unstimulated (A, D), TLR9 agonist CpG-A ODN2216 activated (B, E) or LPS stimulated (C, F) bulk splenocytes (A-C) or B cells (D-F) from EBI2 WT (white bars) or KO (shaded bars) mice towards 7α,25-OHC (indicated concentrations) or 25-OHC (10−6 M). Starting cells and migrating cells were analyzed by flow cytometry. B cell migration was determined by specific staining in the bulk splenocyte population. Data shown are representative of two separate experiments. (TIF) [file pone.0083457.s001.tif]

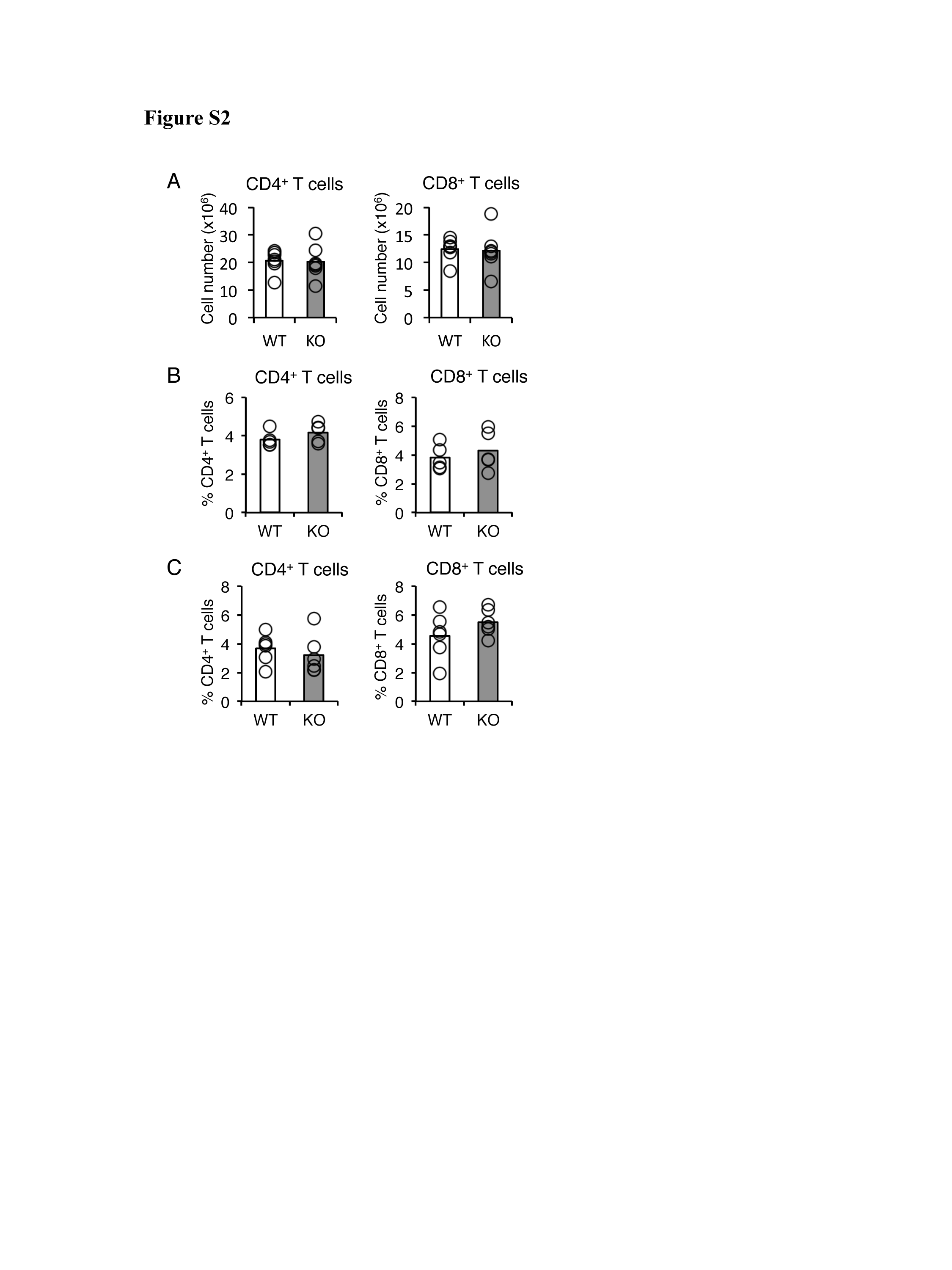

Supplement: Figure S2 — CD4+ and CD8+ T cell distribution in Ebi2 −/− and wild-type mice. A. T cell distribution in spleen of naïve WT and Ebi2 −/− mice. Flow cytometric analysis was used to enumerate CD4+ and CD8+ T cells in spleens of WT (white bars) or Ebi2 −/− (shaded bars) mice (n = 7 per group). B. T cell frequencies in peritoneal lavage fluid of naïve WT and Ebi2 −/− mice (n = 5 per group). C. T cell distribution in peritoneal lavage fluid of Imiquimod-challenged mice 4 hr after i.p. injection (n = 6 per group). Bars represent mean values; white denotes WT, shaded denotes KO; circles represent individual animals. (TIF) [file pone.0083457.s002.tif]

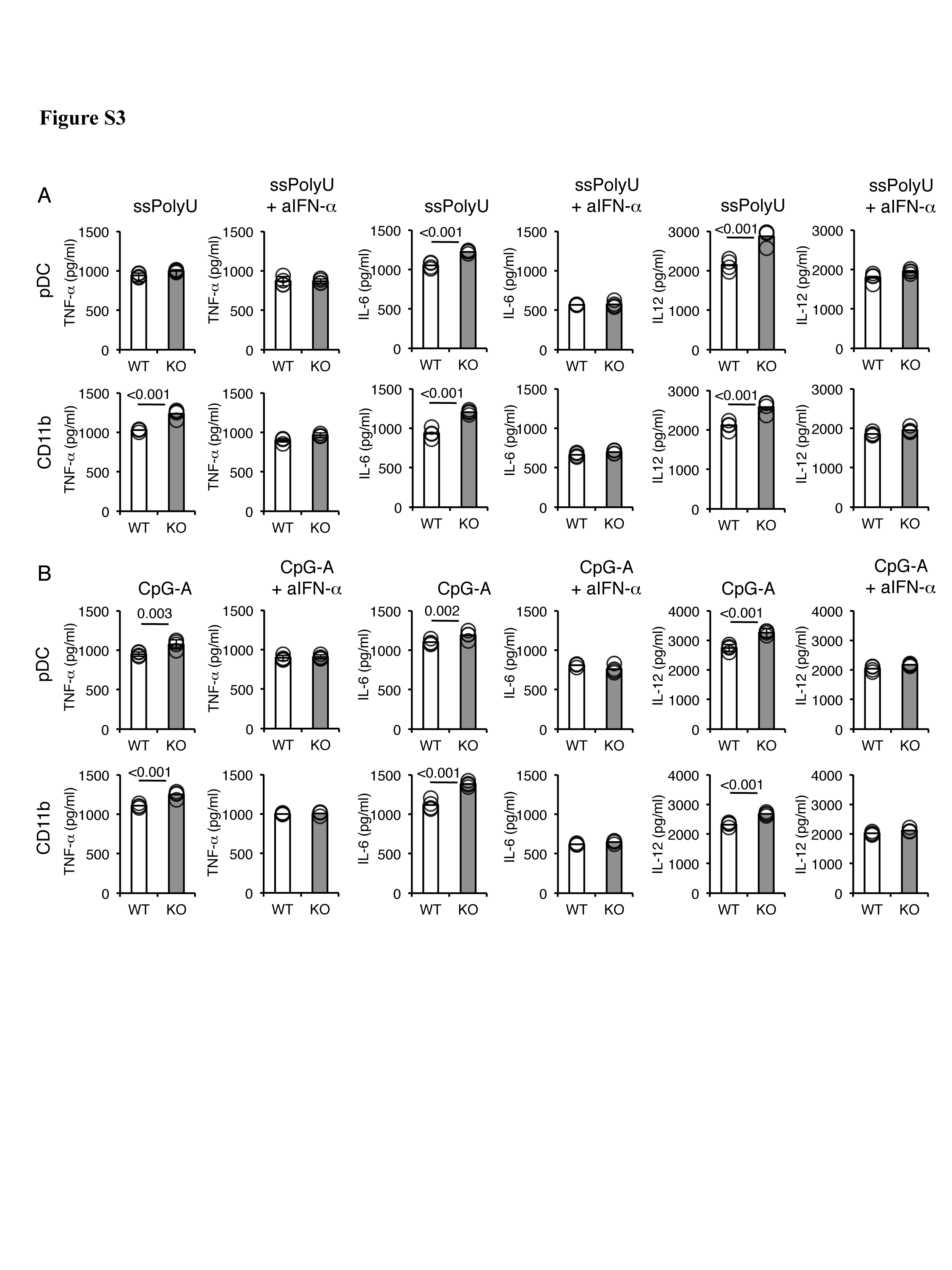

Supplement: Figure S3 — Elevated pro-inflammatory cytokine responses by EBI2-deficient pDCs and monocytes/macrophages activated with TLR7 or TLR9 agonists are due to IFN-α-mediated secondary signaling. pDCs and CD11b+ cells from Ebi2 −/− or WT littermate mice were pre-incubated in the absence or presence of anti-IFN-α mAb, then stimulated with TLR7 agonist ssPolyU (A) or TLR9 agonist CpG-A ODN2216 (B). TNF-α IL-6 and IL-12 in culture supernatants after 40 hr stimulation were measured by ELISA. Cells were purified from pools of 2 spleens each. 8 mice from each group were used, resulting in 4 pools. Bars represent mean values (white denotes WT, shaded denotes KO); circles represent each pool. P-values are denoted when considered statistically significant (p<0.05). (TIF) [file pone.0083457.s003.tif]
