# Supplementary material for: EBI2 Is a Negative Regulator of Type I Interferons in Plasmacytoid and Myeloid Dendritic Cells
Source: PLoS One. 2013 Dec 26;8(12):e83457. doi: 10.1371/journal.pone.0083457 (PMC3873289; doi:10.1371/journal.pone.0083457)
Supplement: Table S1 — Immune cell populations from various tissues in EBI2-deficient mice. (DOC) [file pone.0083457.s004.doc]

**Table S1. Immune cell populations from various tissues in EBI2-deficient mice^1^**.

|  | **spleen** | **MLN** | **PP** | **PerC** | **thymus** | **BM** | **blood** |
| --- | --- | --- | --- | --- | --- | --- | --- |
| **total cell number** | normal |  |  | normal | normal | normal | normal |
| **CD4^+^ T cell** | normal | normal | decrease^5^ | normal | normal | normal | normal |
| **CD4^+^ memory** | normal | normal | decrease^6^ | normal |  | normal | normal |
| **CD4^+^CD25^+^ Treg** | normal | normal | decrease^7^ | normal |  | normal | normal |
| **CD8^+^ T cell** | normal | normal | normal | normal | normal | normal | normal |
| **CD8^+^ memory** | normal | normal | normal |  |  | normal | normal |
| **total B cells** | normal | normal | normal | normal |  | normal | normal |
| **conventional B** | normal |  | normal |  |  |  |  |
| **germinal center B** | normal |  | normal |  |  |  |  |
| **plasma cells** | normal |  |  |  |  | normal |  |
| **follicular B** | normal |  |  |  |  |  |  |
| **marginal zone B** | normal |  |  |  |  |  |  |
| **B1** |  |  |  | normal |  |  |  |
| **pro B + early pre B** |  |  |  |  |  | normal |  |
| **late pre B + immature B** |  |  |  |  |  | normal |  |
| **myeloid DC** | decrease^2^ | decrease^4^ |  | decrease^8^ |  |  | normal |
| **plasmacytoid DC** | decrease^3^ | normal |  | decrease^9^ |  |  | normal |
| **immature DC** | normal | normal |  |  |  |  |  |
| **monocytes/macrophages** | normal | normal |  |  |  |  |  |
| **CD4 SP** |  |  |  |  | normal |  |  |
| **CD8 SP** |  |  |  |  | normal |  |  |
| **CD4^-^CD8^-^ DN** |  |  |  |  | normal |  |  |
| **CD4^+^CD8^+^ DP** |  |  |  |  | normal |  |  |

^1^ Tissues harvested from 8 wk old female EBI2 knockout and WT littermate mice. MLN, mesenteric lymph node; PP, Peyer’s patches; PerC, peritoneal cavity; BM, bone marrow. Absolute cell numbers for spleen, PerC, omentum, thymus, bone marrow and blood (per ml); cell frequencies for MLN, PP. (N = 7 per group for all tissues except PerC and omentum, N = 4 per group).

^2^*P* < 0.0001; ^3^*P* = 0.0262; ^4^*P* = 0.0010; ^5^*P* =0.0004; ^6^*P* = 0.0077; ^7^*P* < 0.0001; ^8^*P* = 0.0152; ^9^*P* = 0.0111; *P*-values determined using mean comparisons with a control using Dunnett’s method.
